# Supplementary material for: Damage Repair versus Aging in an Individual-Based Model of Biofilms
Source: mSystems. 2020 Oct 13;5(5):e00018-20. doi: 10.1128/mSystems.00018-20 (PMC7567578; doi:10.1128/mSystems.00018-20)
Supplement: TEXT S1 [file mSystems.00018-20-s0001.docx]

## Supplementary Materials and Methods

### Determining biofilm structure

The study by Picioreanu *et al.* (1998) (1) was the first to show that biofilm structure is determined by the ratio of potential maximal growth rate to the potential maximal mass transport rate. They called this new dimensionless number the $G$ (Growth) group:

$G=\frac{potential maximal growth rate}{potential maximal mass transport rate}=\frac{\mu_{max}\rho l_{z}^{2}}{D_{G}S_{bulk}}$,

Where $\mu_{max}$ is the maximal specific growth rate, $\rho$ the biomass density, $l_{z}$ the height of the computational domain (length in z direction), $D_{G}$ the diffusion coefficient of the growth substrate and $S_{bulk}$ the substrate concentration in the bulk liquid.

If growth potential is higher than transport potential, growth is limited by transport, so a high $G$ means transport limitation. Note that the length scale in G is that of the vertical computational domain size (z axis) as there was no boundary layer implemented in the model of Picioreanu *et al.* (1998) (1).

Nadell *et al.* (2010) (2) introduced the similar dimensionless group $\delta$, as a measure of the thickness of the actively growing layer at the top of the biofilm that has sufficient access to substrate, given by:

$\delta=\sqrt{\frac{S_{bulk}D_{G}Y_{\mu}}{\mu_{max}\rho b_{L}^{2}}}$,

Where $Y_{\mu}$ is the biomass yield and the length scale, $b_{L}$, is the height of the boundary layer. Basically, $\delta$ is the square root of the reciprocal of $G$, with the yield considered and the length scale changed to the boundary layer height.

We decided that it is better to directly use the ratio of the two rates, as in Picioreanu *et al.* (1998) (1), rather than the square root, as in Nadell *et al.* (2010) (2), so decided to use $\delta^{2}$:

$\delta^{2}=\frac{S_{bulk}D_{G}Y_{\mu}}{\mu_{max}\rho b_{L}^{2}}$.

The differences with the original $G$ group are the introduction of the yield and using the length of the boundary layer instead of the whole height domain. Also, the ratio is ‘reciprocal to $G$’, *i.e.*,

$\delta^{2}=\frac{potential maximal mass transport rate into biofilm}{potential maximal biomass growth rate}$.

If $\delta^{2}$ is high, potential mass transport is higher than potential growth, so growth is not limited by mass transport, and it is a growth limited regime. If $\delta^{2}$ is low, it is a transport limited regime.

We have altered $\delta^{2}$ by lowering $S_{bulk}$, which leads to a lower $\delta^{2}$, which means a more transport limited regimen.

### Measuring biofilm structure

A suitable measure of biofilm structure is the absolute deviation of biofilm front points from the mean front position, $\sigma_{f}$, introduced by Murga *et al.* (1995) (3):

$\sigma_{f}=\frac{\sum_{x=1}^{L} |x-\bar{x}_{f}|\cdot\bar{c}_{f,x}}{\sum_{x=1}^{L} \bar{c}_{f,x}}$,

where $f$ is the grid index of the biofilm front or the biofilm liquid interface,$L$, the number of grid elements in the vertical direction $x$, $\bar{x}_{f}=\frac{\sum_{x=1}^{L} x\cdot\bar{c}_{f,x}}{\sum_{x=1}^{L} \bar{c}_{f,x}}$ the weighted mean front position, $\bar{c}_{f,x}=\frac{1}{N}\sum_{y=1}^{N} c_{f,y,x}$ the mean density of front points at distance $x$ from the substratum (solid surface to which the biofilm is attached), $c_{f,y,x}$ the state of the occupation matrix (1 for a front point, 0 otherwise) and $N$ the number of grid elements in the horizontal direction $y$. This metric depends on the mean thickness of the biofilm.

Biofilm front points are those biofilm grid elements that have at least one neighboring grid element that is not part of the biofilm or the substratum to which the biofilm is attached. Note that agents are spherical particles with continuous positions rather than grid elements in iDynoMiCS, so agent positions and masses are mapped into a biomass density grid for this analysis.

### References

1. Picioreanu C, van Loosdrecht M, Heijnen J. 1998. Mathematical Modeling of Biofilm Strucutre with a Hybrid Differential-Discrete Cellular Automata Approach. Biotechnol Bioeng 58:101–116.

2. Nadell CD, Foster KR, Xavier JB. 2010. Emergence of spatial structure in cell groups and the evolution of cooperation. PLoS Comput Biol 6.

3. Murga R, Stewart PS, Daly D. 1995. Quantitative analysis of biofilm thickness variability. Biotechnol Bioeng 45:503–510.
