# Supplementary material for: Damage Repair versus Aging in an Individual-Based Model of Biofilms
Source: mSystems. 2020 Oct 13;5(5):e00018-20. doi: 10.1128/mSystems.00018-20 (PMC7567578; doi:10.1128/mSystems.00018-20)
Supplement: TABLE S1 [file mSystems.00018-20-st001.docx]

**TABLE S1A**

|  | **Initial cell density** | **Biomass ^1^** | | | **Population size ^1^** | | | **Growth rate ^1^** | | |
| --- | --- | --- | --- | --- | --- | --- | --- | --- | --- | --- |
|  |  | ***Sp1*** | ***Sp2*** | ***P-value ^2^*** | ***Sp1*** | ***Sp2*** | ***P-value ^2^*** | ***Sp1*** | ***Sp2*** | ***P-value ^2^*** |
| ***AR vs DS*** *(Sp1 = AR, Sp2 = DS) ^3^* | ***4*** | 31 | 19 | 0.119 | 28 | 22 | 0.480 | 31 | 19 | 0.119 |
|  | ***8*** | 38 | 12 | 0.000306 | 38 | 12 | 0.000306 | 37 | 13 | 0.000936 |
|  | ***16*** | 48 | 2 | 0.00 | 48 | 2 | 0.00 | 48 | 2 | 0.00 |
|  | ***32*** | 50 | 0 | 0.00 | 50 | 0 | 0.00 | 50 | 0 | 0.00 |
| ***AR vs AR*** *(Sp1 = AR, Sp2 = AR) ^3^* | ***4 ^4^*** | 22 | 28 | 0.480 | 21 | 29 | 0.322 | 19 | 31 | 0.119 |
|  | ***8*** | 27 | 23 | 0.672 | 27 | 23 | 0.672 | 30 | 20 | 0.203 |
|  | ***16*** | 23 | 27 | 0.672 | 23 | 27 | 0.672 | 24 | 26 | 0.888 |
|  | ***32*** | 24 | 26 | 0.888 | 23 | 23 | 0.672 | 25 | 25 | 1.00 |
| ***DS vs DS*** *(Sp1 = DS, Sp2 = DS) ^3^* | ***4 ^4^*** | 28 | 22 | 0.480 | 29 | 21 | 0.322 | 35 | 15 | 0.00660 |
|  | ***8*** | 24 | 26 | 0.888 | 24 | 26 | 0.888 | 25 | 25 | 1.00 |
|  | ***16*** | 19 | 31 | 0.119 | 19 | 31 | 0.119 | 20 | 30 | 0.203 |
|  | ***32*** | 27 | 23 | 0.672 | 27 | 23 | 0.672 | 29 | 21 | 0.322 |

^1^ Number of competitions (50 in total) won by each strategy when determined by three different metrics: total biomass, size of the populations or population growth rate at the end (see Figs. 6 and S7 for time courses).

^2^ *P*-values shown are from the results of a proportion test carried out in Python, as the competitions have two potential outcomes and are therefore binomially distributed. All *p*-values are shown to 3 significant figures and statistically significant differences between competitiveness of strategies are indicated with yellow shading.

^3^ For adaptive repair *vs* damage segregation, species 1 (Sp1) is adaptive repair and species 2 (Sp2) is damage segregation. For the two controls, both species have the same strategy.

^4^ Note that growth rate appears to be significantly different in the 4 cell control where two identical strategies compete, therefore, final growth rate is not a reliable metric for fitness.

**TABLE S1B**

| **Parameter** | **Original value** | **Lower value** | **Higher value** | **Relationship to previous value and justification for use** |
| --- | --- | --- | --- | --- |
| $\mu_{max}$ (h^-1^) | 1.2 | 0.6 | 2.4 | Half/double previous value. Highest specific growth rates measured for *E. coli* are 2.5 h^-1^ (107). |
| $Y_{\mu}$ (g g^-1^) | 0.444 | 0.222 | 0.888 | Half/double previous value. The growth yield for budding yeast can be between 0.1 and 0.6 g g^-1^ substrate (108). |
| $Y_{r}$ (g g^-1^) | 0.8 | 0.444 | 1 | It is assumed that repair is more efficient than generating new material, so this is constrained between $Y_{\mu}$ (0.444 g g^-1^) and 1, equivalent to 100% efficiency, meaning no cost to carrying out repair. |
| $P_{div}$ (radius; µm) | 0.8 | 0.37 | 1.72 | Divide/multiply protein mass by ten. Equivalent to cells of mass 431, 43.1 and 4310 fg, respectively. Some of the smallest cells found have an equivalent spherical diameter of 0.25 µm (109) while the budding yeast can be up to 8,000 fg (110). |
| $S_{bulk}$ (g L^-1^) | 0.003556 | 0.0003556 | 0.03556 | Divide/multiply previous substrate concentration by ten, equivalent to substrate concentrations of 0.03556%, 0.3556% and 0.003556% (w/v), respectively. |
| $K_{S}$ (g L^-1^) | 0.00234 | 0.0000534 | 0.108 | Highest (111) and lowest (112) experimental values for *E. coli* substrate affinity. |
| $a'$ (h^-1^/$\mu_{G}$) | 0.22 | 0.11 | 0.44 | Half/double previous value. |

**TABLE S1C**

| **KEGG ortholog** | **Genes** | **Organism/Function** |
| --- | --- | --- |
| K04043 | dnaK (Hsp70) | *Escherichia coli*  Prevention of aggregation, reactivation of aggregated proteins in cooperation with clpB (Hsp104) and folding of newly synthesized proteins and misfolded protein species (1). |
| K03686 | dnaJ (Hsp 40) |  |
| K05516 | cbpA | *E. coli*  Can compensate for dnaJ if this is impaired/missing (2). |
| K03695 | clpB, Hsp104 | *E. coli*, *Saccharomyces cerevisiae*  Reactivation of aggregated proteins in cooperation with an Hsp70 chaperone system (1). |
| K04080 | ibpA (sHSP) | *E. coli*  Small heat shock proteins responsible for prevention of irreversible protein aggregation (1). |
| K04081 | ibpB (sHSP) |  |
| K04077 | Hsp60 | Archaea  Prevent the accumulation of misfolded conformers (1). Hsp60s are contained by all archaea, while only a subset include others such as Hsp70 and Hsp40 (3). |

**TABLE S1D**

| **Organism** | **Number of assemblies^1^** | **Median genome (Mb)^1^** | **Median protein count ^1^** |
| --- | --- | --- | --- |
| *Escherichia coli* | 20,025 | 5.13 | 4,764 |
| *Staphylococcus aureus* | 11,544 | 2.84 | 2,725 |
| *Salmonella enterica* | 11,277 | 4.80 | 4,446 |
| *Klebsiella pneumoniae* | 9,183 | 5.59 | 5,349 |
| *Streptococcus pneumoniae* | 8,585 | 2.09 | 2,010 |
| *Mycobacterium tuberculosis* | 6,568 | 4.38 | 4,101 |
| *Pseudomonas aeruginosa* | 5,210 | 6.61 | 6,105 |
| *Acinetobacter baumannii* | 4,514 | 3.97 | 3,693 |
| *Listeria monocytogenes* | 3,344 | 2.97 | 2,933 |
| Uncultured *Pelagibacteraceae* spp. | 2,364 | 0.82 | Not available |

^1^ Values were taken from the NCBI database on 1 June 2020

**TABLE S1E**

| **JGI genome ID** | **Prokka** | | | | | | **Repair genes total** | **CheckM** | | |
| --- | --- | --- | --- | --- | --- | --- | --- | --- | --- | --- |
|  | **dnaK** | **dnaJ** | **cpbA** | **clpB** | **ibpB** | **Hsp60** |  | **Completeness (%)** | **Contamination (%)** | **Taxonomy** |
|  | **COG0443** | **COG0484** | **COG2214** | **COG0542** | **COG0071** | **COG0459** |  |  |  |  |
| 2510065005 | 0 | 0 | 0 | 0 | 0 | 2 | 2 | 98.02 | 1.98 | p__Euryarchaeota |
| 2600255021 | 1 | 0 | 0 | 0 | 0 | 0 | 1 | 63.81 | 0.57 | f__Pasteurellaceae |
| 2617271188 | 1 | 1 | 0 | 1 | 0 | 0 | 3 | 49.38 | 0.94 | c__Betaproteobacteria |
| 2639762705 | 0 | 0 | 0 | 1 | 0 | 1 | 2 | 61.88 | 0.00 | p__Proteobacteria |
| 2671180212 | 0 | 0 | 0 | 1 | 1 | 1 | 3 | 66.71 | 0.53 | f__Rhodobacteraceae |
| 2681812881 | 0 | 0 | 0 | 0 | 0 | 1 | 1 | 38.14 | 0.00 | c__Gammaproteobacteria |
| 2684622808 | 0 | 0 | 0 | 0 | 0 | 0 | 0 | 42.26 | 1.78 | g__Staphylococcus |
| 2700988635 | 0 | 0 | 0 | 0 | 0 | 0 | 0 | 47.30 | 0.00 | p__Proteobacteria |
| 2706794582 | 1 | 0 | 0 | 2 | 0 | 1 | 4 | 50.47 | 1.77 | o__Lactobacillales |
| 2716884108 | 0 | 1 | 0 | 3 | 0 | 0 | 4 | 74.05 | 5.80 | k__Bacteria |
| 2718217655 | 0 | 0 | 0 | 0 | 0 | 2 | 2 | 97.47 | 0.63 | c__Thermoprotei |
| 2671180212 ^1^ | 1 | 1 | 0 | 2 | 0 | 0 | 4 | NA | NA | g__Roseobacter |
| 638154507 | 0 | 0 | 0 | 0 | 0 | 1 | 1 | 65.06 | 0.00 | k__Bacteria |
| 649990008 | 1 | 1 | 0 | 1 | 0 | 1 | 4 | 99.34 | 2.65 | c__Bacilli |
| 2236876029 | 0 | 1 | 0 | 1 | 0 | 1 | 3 | 98.91 | 3.23 | f__Bacillaceae |
| 2537562134 | 1 | 2 | 0 | 2 | 0 | 1 | 6 | 96.74 | 1.60 | p__Euryarchaeota |
| 2728369649 | 1 | 1 | 0 | 1 | 0 | 1 | 4 | 100.00 | 0.00 | g__Streptococcus |

^1^ For genome 2671180212, annotations and taxonomy were downloaded from the JGI IMG genome database as the complete genome was not available.
